# Molecular Assessment of Epiretinal Membrane: Activated Microglia, Oxidative Stress and Inflammation

**DOI:** 10.3390/antiox9080654

**Published:** 2020-07-23

**Authors:** Sushma Vishwakarma, Rishikesh Kumar Gupta, Saumya Jakati, Mudit Tyagi, Rajeev Reddy Pappuru, Keith Reddig, Gregory Hendricks, Michael R. Volkert, Hemant Khanna, Jay Chhablani, Inderjeet Kaur

**Affiliations:** 1 Prof Brien Holden Eye Research Centre, LV Prasad Eye Institute, Hyderabad 500034, India; svishwakarma17@gmail.com (S.V.); rkgupta@iimcb.gov.pl (R.K.G.); 2 Manipal Academy of Higher Education, Manipal, Karnataka 576104, India; 3 Ophthalmic Pathology Laboratory, L.V. Prasad Eye Institute, Hyderabad 500034, India; saumyajakati@lvpei.org; 4 Smt. Kanuri Santhamma Retina Vitreous Centre, L.V. Prasad Eye Institute, Hyderabad 500034, India; drmudit@lvpei.org (M.T.); rajeev@lvpei.org (R.R.P.); 5 Department of Radiology, University of Massachusetts Medical School, Worcester, MA 01655, USA; keith.reddig@umassmed.edu (K.R.); Gregory.Hendricks@umassmed.edu (G.H.); 6 Department of Microbiology & Physiological Systems, University of Massachusetts Medical School, Worcester, MA 01655, USA; michael.volkert@umassmed.edu; 7 Department of Ophthalmology & Visual Sciences, University of Massachusetts Medical School, Worcester, MA 01655, USA; hemant.khanna@umassmed.edu

**Keywords:** human retina, epiretinal membrane, internal limiting membrane, vitreoretinal surgery, macular hole, proliferative diabetic retinopathy

## Abstract

Fibrocellular membrane or epiretinal membrane (ERM) forms on the surface of the inner limiting membrane (ILM) in the inner retina and alters the structure and function of the retina. ERM formation is frequently observed in ocular inflammatory conditions, such as proliferative diabetic retinopathy (PDR) and retinal detachment (RD). Although peeling of the ERM is used as a surgical intervention, it can inadvertently distort the retina. Our goal is to design alternative strategies to tackle ERMs. As a first step, we sought to determine the composition of the ERMs by identifying the constituent cell-types and gene expression signature in patient samples. Using ultrastructural microscopy and immunofluorescence analyses, we found activated microglia, astrocytes, and Müller glia in the ERMs from PDR and RD patients. Moreover, oxidative stress and inflammation associated gene expression was significantly higher in the RD and PDR membranes as compared to the macular hole samples, which are not associated with inflammation. We specifically detected differential expression of hypoxia inducible factor 1-α (*HIF1-α*), proinflammatory cytokines, and Notch, Wnt, and ERK signaling pathway-associated genes in the RD and PDR samples. Taken together, our results provide new information to potentially develop methods to tackle ERM formation.

## 1. Introduction

The retina is a multilayered light-sensing neural tissue located at the back of the eye [1]. The outer retina is composed of photoreceptors, which convert light into electrical signals and transmit them to the inner neurons and to the ganglion cells in the inner retina. The ganglion cell layer is also composed of Muller glia, whose foot processes are embedded into a thin transparent layer in the basal lamina of the inner retina called the inner limiting membrane (ILM). The ILM forms a boundary between the retina and the underlying vitreous [2,3,4].

Epiretinal membrane (ERM), a fibrocellular tissues forms on the surface of ILM, mainly on the macula. ERMs can be either: (a) idiopathic or (b) associated with ocular inflammatory diseases such as diabetic retinopathy and retinal detachment [5,6]. ERMs distort the underlying ILM and alter retinal morphology and function. Survival and proliferation of the cells present on the ERM also depends upon the oxygen level. Ischemic conditions lead to accumulation/activation of the transcription factor, hypoxia-inducible factor (HIF-1α), which causes secretion of vasoactive cytokines vascular leakage and macular edema [7]. Growth factors such as hepatocyte growth factor (HGF), heparin-binding epidermal growth factor (HB-EGF), and epidermal growth factor (EGF) others and other inflammatory molecules released due to any retinal insults also induce the migration of retinal cells like RPE to the vitreous to proliferate and induce the retinal membrane formation. 

Currently, ILM removal along with ERM peeling is considered the gold standard treatment and results in minimal recurrence of the ERMs. However, the extent of successful restoration of vision varies as ERM peel can lead to retinal breaks and perturb outer retina function. Therefore, ILM peeling requires a decision on the inevitability of ERM removal only for selected needful patients. 

We propose that understanding the cellular composition and gene expression signature of the ERMs will assist in designing strategies to tackle ERM formation with minimal effects on the ILM or avoid ERM peeling in patient where it may play a beneficial role, such as in preventing macular hole formation [8,9]. Previous remarkable studies have identified hyalocytes, glial cells, retinal pigment epithelial cells, fibrocytes and myofibroblast along with non-cellular component like fibronectin, and actin [10,11]. However, a comprehensive analysis of the different components and a gene expression profile related to oxidative stress and pro-inflammatory signaling associated genes have not been investigated.

In the present study, we carried out a comparative histological, ultrastructural and gene expression analysis of fibrocellular membranes associated with inflammatory conditions of the eye. Our results expand our understanding on the role of activated microglia in ERMs and suggest that immunomodulation by targeting microglia could be a potential therapy for a better clinical management of the condition.

## 2. Materials and Methods

### 2.1. Enrollment of Subjects

Initially, ILM specimens (*n* = 30) were collected during vitreo-retinal surgery from June 2016 to September 2017 at the L.V. Prasad Eye Institute by a single vitreoretinal surgeon (Jay Chhablani) and were used for Hematoxylin and Eosin (H&E), transmission electron microscopy (TEM) and immunohistochemistry (IHC)-based evaluations. Additional membranes (*n* = 15, 3 in MH, 8 in PDR and 4 in RD) were collected from the additional ORs of two other vitreo-retinal surgeons (Mudit Tyagi and Rajeev Reddy Pappuru) for the targeted gene expression profiling. This prospective study was approved (Ethic Ref No. LEC 02-14-029) by the Institutional Review Board (IRB) of L.V. Prasad Eye Institute, Hyderabad, India (LVPEI) and adhered to the tests of the Declaration of Helsinki. A prior informed consent was obtained from each study subject. All subjects underwent a comprehensive ophthalmic examination with Snellen’s visual acuity, slit lamp examination, intraocular pressure measurement using applanation tonometry and dilated fundus examination. All eyes except eyes with RRD underwent preoperative spectral domain optical coherence tomography (OCT) Cirrus High Definition-OCT (Carl Zeiss Meditec, Dublin, CA, USA). Five-line high definition raster scan was performed.

The membranes removed as part of the routine surgical management were collected from different pathological conditions. Subjects with the diagnosis of vitreoretinal interface disorders including idiopathic macular hole (MH), proliferative diabetic retinopathy (PDR) and rhegmatogenous retinal detachment (RD) who underwent pars plana vitrectomy (PPV) with Brilliant blue-G (BBG)—assisted inner limiting membrane peeling (ILM peeling) were included in the study.

### 2.2. Surgical Details

Surgical procedure included 23- or 25-gauge vitrectomy. After induction of posterior vitreous detachment, vitrectomy was completed. Epiretinal membranes were removed using end-gripping forceps. For ILM staining, 0.02% brilliant blue was used under air infusion. After one minute of staining, ILM at the macular area was removed using ILM forceps. In eyes undergoing macular hole repair, C3F8 gas (12–15%) was used as tamponade. Cataract surgery was performed during the follow-up in cases where it was needed with standard phacoemulsification procedure, and posterior chamber intraocular lens was implanted. In cases with rhematogenous RD, brilliant blue staining of ILM was performed under fluid with infusion canula off.

### 2.3. Histopathological Investigation 

All surgically peeled membrane samples were embedded in the optimal cutting temperature compound (OCT)-medium (Leica Biosystems, Nussloch GmbH, Germany) and stored at −20 °C initially for slow freezing. After freezing, sections of 4 µm thickness were prepared using cryostat (Leica CM1950 Clinical Cryostat-Leica Biosystems, Nussloch GmbH, Germany) for each subject. The sections were stained with hematoxylin and eosin (H&E) dyes and images were taken at 4× and 10× with an inverted microscope (Olympus Corporation, Shinjuku, Tokyo, Japan).

### 2.4. Ultrastructural Evaluations of Membranes Using Transmission Electron Microscopy

The surgically excised membranes were stored in 3% glutaraldehyde at room temperature for 24 h followed by the standard procedures [11,12] for sample preparation. Ultra-thin (70 nm) sections were made with a diamond knife on ultramicrotome (Leica Ultra cut UCT-GA-D/E-1/00), mounted on gold grids and stained with saturated aqueous uranyl acetate (UA) and counter stained with Reynolds lead citrate (LC) and viewed under TEM (Model: CM10,Philips, North Worcester, MA, USA).

### 2.5. Cellular Characterization Using Immunohistochemistry

The tissue sections were processed for immunohistochemistry using cell type specific primary antibodies including rabbit anti-glial fibrillary acid protein antibody (GFAP) (glial marker, 1:400, Z0334, Dako, Glostrup, Denmark), rabbit anti-ALDH1L1 (astrocytes, 1:500, ab190298, Abcam, Cambridge, MA, USA), mouse anti-cellular retinaldehyde-binding protein (CRALBP) (retinal astrocytes, 1:80, ab15051, Abcam), rat anti-F4/80 (macrophages, 1:30, ab16911, Abcam,), and rabbit anti-oxidation resistance 1 (Oxidative stress, 1:100, HPA027395,Sigma, St. Louis, MO, USA), against OXR1; a protein that controls the resistance of neuronal cells to oxidative stress. After sectioning the slides were stored at −20 °C. For the experiment, slides were taken out from the −20 °C and kept at room temperature for 30 min to bring them to room temperature. Next, the slides were dipped in fixative (chilled methanol: acetone (95:5) solution) for 15 min and dired for 30 min at room temperature. The slides were then washed three times with 1× PBS. 1% BSA (Himedia, Lab Pvt. Ltd., Mumbai, Maharashtra, India) and incubated with blocking buffer containing 0.1%-Tween20 (Fisher Scientific, Guldensporenpark, Merelbeke, Belgium) for 2 h. The sections were then incubated with the primary antibody overnight. The primary antibodies were diluted in 0.1% blocking buffer. After overnight incubation, the slides were washed three times with 1X PBS, for 5 min each, followed by secondary antibody incubation at a dilution 1:300 (Alexa Fluor^®^ 488 Goat Anti-Rabbit IgG, (A-11008, Invitrogen, Waltham, MA, USA) and Alexa Fluor^®^ 488 Goat Anti-Mouse IgG, (ab150117, Abcam). Negative control IHC was done to test the specificity of antibody and to identify false positive results shown in Figure S1. Details of the antibodies are provided in Table S1.

### 2.6. Imaging and Image Analysis

The digital inverted microscope (EVOS FL Cell Imaging System, Life Technology, Paisley, UK) was used for the imaging at X200 final magnification. Further, total number of cells based on DAPI staining and the total number of positively stained cells for each marker were counted on individual sections corresponding to different pathological conditions. Average and standard deviation of the mean number of cells for each marker from specimens (*n* = 3) in different pathological conditions were calculated. Percentage of OXR1 positive cells was calculated using: (total number of OXR1 positive cells divided by total number of DAPI stained cells) × 100.

### 2.7. Targeted Gene Expression Profiling by qRT-PCR

Total RNA was isolated from membranes using PureLink™ RNA Mini Kit (Invitrogen™, catalog no. 12183018A) following the manuals as instructed. RNA was converted to cDNA using verso cDNA synthesis kit (Thermo Scientific™, Waltham, MA, USA, catalog no. AB1453B). A reaction mixture of volume 10μL was made using iTaq™ Universal SYBR^®^ Green Supermix (BIO-RAD, Hercules, CA, USA, catalog no. 38220090), 200nM of primer and cDNA and further, subjected to Applied Biosystems 7900 HT system. A relative measurement of the concentration of target gene (CT) was calculated by using software SDS 2.4 (Applied Biosystems, Foster City, CA, USA). Differential gene expression was analyzed using the 2^−ΔΔ*C*^_T_ method. Statistical analyses were performed using the 2^−ΔΔ*C*^_T_ ± SEM. *β-actin*; a housekeeping gene was used as a normalizing control. The primer sequences used for semi-quantitative real time PCRs are given in the Table S2.

### 2.8. Statistical Analysis

The total number of cells were counted manually in a defined fixed quadrant and equal area of each images. Average number of the cells were accounted for further the descriptive statistical analysis included mean and standard deviation (SD) described in Section 2.6. *t*-Test was used to calculate significance for qPCR data and mean fold changes ± SE were plotted in a graph. A *p*-value less than 0.05 was taken as statistically significant.

### 2.9. Bioinformatic Analysis

For gene expression analysis, a heat map was generated using the online tool Heatmapper (http://www.heatmapper.ca/expression/). Protein-protein interaction was predicted using STRING (v 11.0) (https://www.string-db.org/). Where network edges were defined based on evidence. The minimum required interaction score was kept at minimum confidence of 0.400, with no more than 50 interactors. The active interaction sources were text mining, experimentally proved, database, co-expression data, neighborhood joining, gene fusion, and co-occurrence.

## 3. Results

### 3.1. Demographical Data

The mean age (mean age ± standard deviation) of the subjects included in this study was 55.22 ± 7.35. The number of males and females were 33 and 12 respectively.

### 3.2. Histological Evaluations and Cellular Profiling

**Figure 1:**
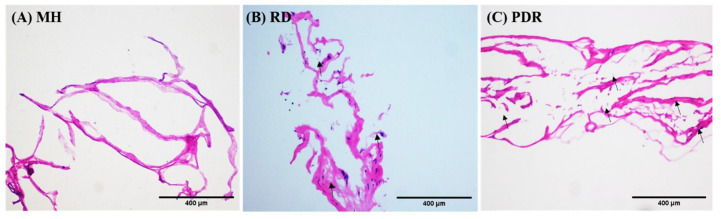
H&E staining of epiretinal membrane (**A**) macular hole, (**B**) retinal detachment, (**C**) proliferative diabetic retinopathy. Photomicrographs in all except macular hole show the presence of spindle shaped cells (black pointed arrows) and the adjacent fibro-collagenous tissue. (H&E; scale bar = 400 μm).

Histological analysis of the ERMs revealed spindle cells with round or oval hyperchromatic nuclei (black arrow; Figure 1 and Figure S1) and pale eosinophilic cytoplasm with or without uveal pigment. The cellularity varied among specimens; it was lower in macular hole samples as compared to PDR and RD. We did not detect a significant difference in cellularity between PDR and RD samples.

### 3.3. Ultrastructural Characterizations

Next, we performed ultrastructural characterization by TEM of the membranes extracted from three subjects belonging to the PDR and MH groups. The membranes from RD cases were not included due to insufficient numbers.

**Figure 2:**
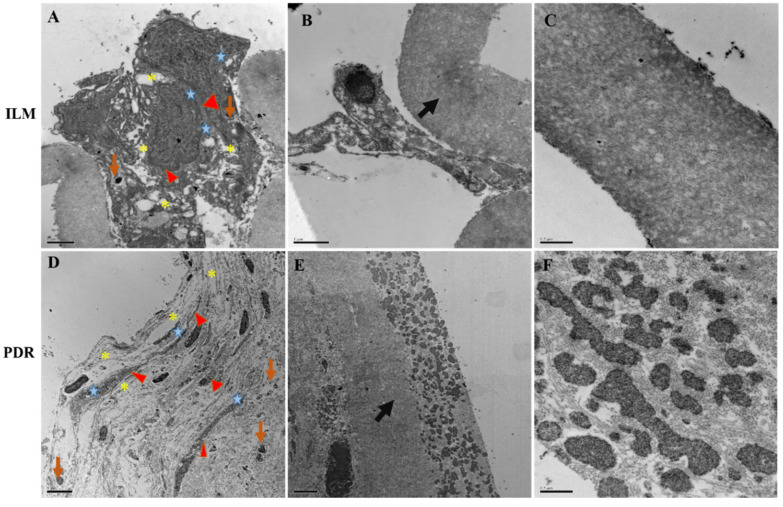
Transmission electron microscopy images of fibrocellular membranes from; (**A**–**C**); macular hole, and (**D**–**E**); proliferative diabetic retinopathy membrane showing the presence of astrocytes, Müller glia processes and microglia. The cell body of a Müller cell can be identified by its nucleus and dark cytoplasm (red color filled triangular shape). An astroglial cell process with flattened intermediate filaments (yellow colored asterisk) can be observed next to the Müller cell. The microglia can be seen with round shaped dark cell bodies (brown arrow) and glial cells processes with skyblue star shaped structure. (**B**,**E**) show collagenous fibrils of membrane indicated by black arrow and; (**C**,**F**) show their corresponding magnified image.

The retinal cell-types were characterized based on previously published morphological details [13,14,15,16,17,18]. Varying thickness of collagen fibrils, the main component of the ERMs was observed in both PDR and MH. We frequently detected astrocytes, Müller glia with long processes, microglia like cells with thick dark heterochomatic cell body and a few dark pigmented cells (Figure 2) in the PDR versus the MH specimens.

### 3.4. Characterization of Different Cell Types in Each Group

We next validated the presence of the specific cell types by indirect immunofluorescence using appropriate marker antibodies. We used nine samples, with three samples from each group (MH, RD and PDR). The MH samples were used as negative controls.

**Figure 3:**
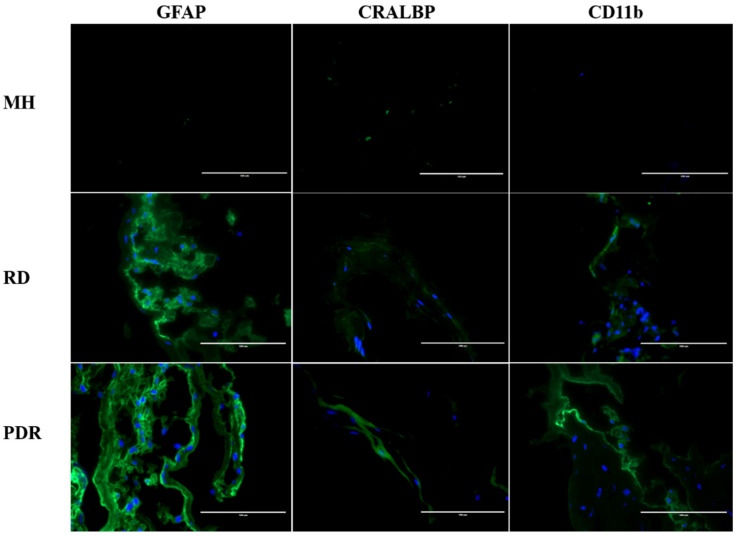
Representative images showing IHC of membranes from pathological conditions MH, RD and PDR with intense cytoplasmic and membranous expression of GFAP, CRALBP, and CD11b proteins. (Scale bar; 100 μm).

Our analyses confirmed the presence of both macroglial and microglial cells along with other proliferative spindle shaped cells. While GFAP and ALDH1L1, markers for astrocytes (and gliosis) were mostly detected in all specimens, the PDR samples showed greater number of GFAP positive cells present in the membrane as compared to MH and RD specimens (Figure 3 and Figure S2). CRALBP staining did not significantly differ in the Müller glia among PDR and RD samples. The number of CD11b (microglia marker) positive cells was higher in the RD membranes as compared to the others.

Quantification of the cells present on the membrane validated the higher number of CD11b positive cells in RD than MH and PDR. On the other hand, increased number of GFAP positive cells was seen in PDR as compared to all cases (Table 1).

### 3.5. Evaluation of Oxidative Stress among Different Pathological Condition

**Figure 4:**
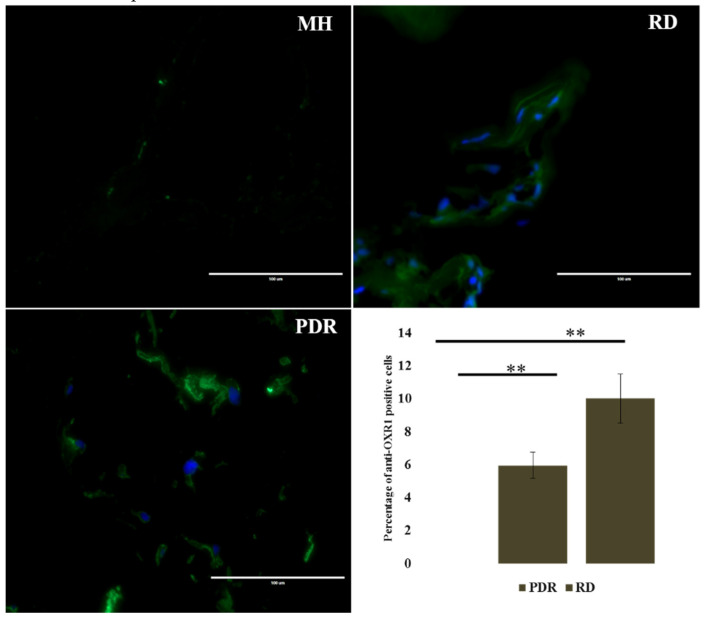
Representative images of IHC showing the expression of OXR1 in MH, RD, and PDR. The graph shows the mean percentage of OXR1 positive cells for each pathological condition (*n* = 3) (scale bar; 100 μm) ** *p*-value < 0.05.

To further explore the involvement of oxidative stress in the ERMs, we performed immunofluorescence analysis using anti-OXR1 antibody in the same tissue specimens (*n* = 3) of MH, RD and PDR cases. OXR1 is expressed at elevated levels in cells undergoing oxidative stress [19]. We found a greater number of OXR1-positive cells in the RD sample than PDR (*p* = 0.04) shown in Figure 4; none of the cells was positive for OXR1 in the membranes from MH.

### 3.6. Gene Expression Analysis of Oxidative Stress and Inflammation-Related Pathway Genes

**Figure 5:**
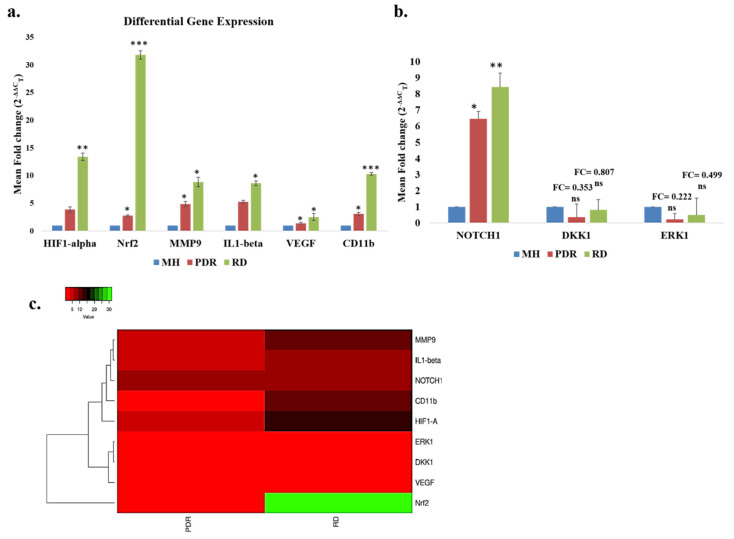
(**a**,**b**) Differential gene expression analysis of oxidative stress, inflammatory markers and their associated pathway involved in the pathogenesis of epiretinal membrane of RD, PDR compared to macular hole (* *p* ≤ 0.05, ** *p* ≤ 0.01, *** *p* ≤ 0.001) (**c**) Heat map showing the expression pattern of the differentially expressed genes in PDR and RD.

Oxidative stress though being closely interlinked to inflammation, could also be the cumulative effect of continued insults to the eye such as ischemia and hyperglycemia [20]. To further quantitate the underlying oxidative stress and inflammation in membrane formation and DR pathogenesis, a targeted gene expression profiling was undertaken by semi-quantitative real time PCR using SYBR green chemistry. We used MH as control and the expression was measured among MH, PDR and RD. Initially, to measure the underlying oxidative stress, we used *Nrf2*, and *HIF1-α* while *MMP9*, and *IL1-β* were used as markers of inflammation. Expression of *CD11b* and *VEGF* was also measured for activated microglia and angiogenesis. In comparison to MH, the gene expression of *Nrf2*, *HIF1-α* and *MMP9* was higher in the RD (*Nrf2*: 31.81 ± 0.76, *** *p* = 0.00066; *HIF1-α*: 13.423 ± 0.63, *** *p* = 0.005; *MMP9*: 8.851 ± 0.86, * *p* = 0.04) followed by PDR (*Nrf2*: 2.76 ± 0.16, ** *p* = 0.007; *HIF1-α*: 3.88 ± 0.47, ns, *p* ≥ 0.05 and; *MMP9*: 4.901 ± 0.86, * *p* = 0.03). Consistently, we noticed increased expression of *IL1-β* in RD (8.648 ± 0.43, * *p* = 0.03) and PDR (5.282 ± 0.27, ns, *p* ≥ 0.05) compared to MH. *VEGF*, an angiogenic marker showed almost similar elevated level of expression in both RD (2.542 ± 0.63, * *p = 0.031*) and PDR (1.42 ± 0.13, * *p* = 0.01). Likewise, *CD11b*, also showed significantly higher expression in RD (10.333 ± 0.23, *** *p* = 0.000076), and PDR (3.092 ± 0.25, * *p* = 0.011) than MH (Figure 5a).

We then analysed the expression of the genes associated with major signaling pathways including *NOTCH1, DKK1* and *ERK1* in the ERMs from RD and PDR. Besides oxidative stress and inflammation, the additional genes and pathway chosen for this analysis were selected based on the existing proposed pathogenic theories in the literature and those known to be involved in retinal cellular proliferation and maintenance. There was significant upregulation of *NOTCH1* gene expression in RD (8.427 ± 0.64, ** *p*-value=0.008) and PDR (6.459 ± 0.81, * *p*-value = 0.02) as compared to control (MH) (Figure 5b). Although *DKK1* and *ERK1* were downregulated, the effect was not statistically significant.

Further, the heatmap, a clustering based method that groups genes and/or samples together based on the similarity of their gene expression pattern, showed the differential expression profile of the genes for PDR and RD shown in Figure 5c. Except for *NRF2* all other genes that clustered together seems to be regulated by common processes.

**Figure 6:**
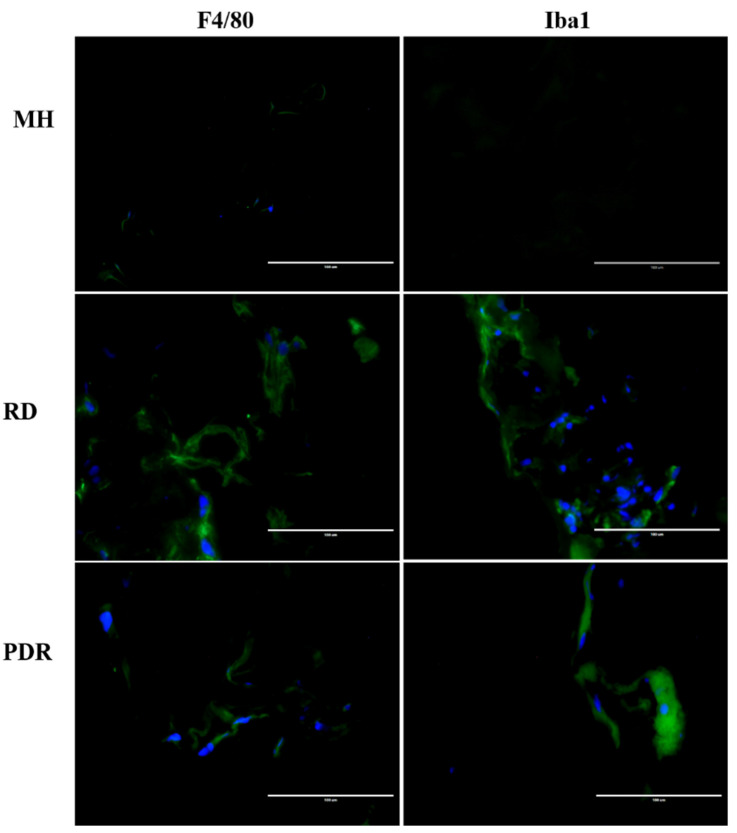
Validation of activation of microglia on the membrane shown by immunohistochemistry using marker for resting; F4/80 and activated stage; Iba1 in different pathological conditions MH, RD and PDR; scale bar; 100 μm.

To further validate the presence of activated microglial, we again performed immunofluorescence analyses of both resting microglia and activated microglia markers: F4/80 and Iba1 respectively. The results showed the presence of both resting as well as activated microglia in all pathologies except MH as shown in Figure 6.

### 3.7. Protein-Protein Interactions for the Differentially Expressed Genes by In-Silico Analysis

**Figure 7:**
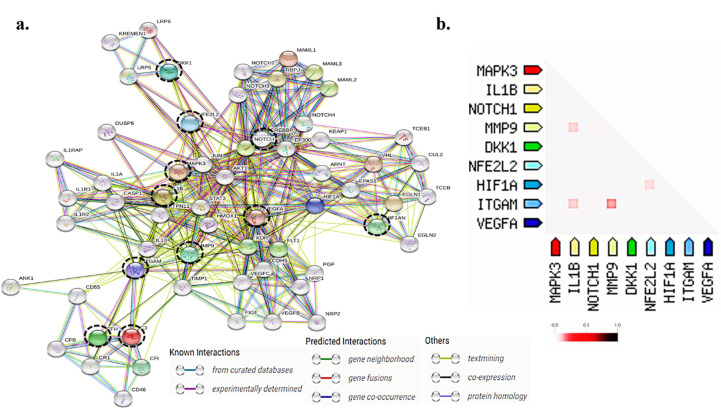
(**a**) Prediction of protein-protein interaction for the differentially expressed genes among PDR and RD in the present study. Protein 3D structure are enclosed in the circles. The colors of bond between the proteins indicate the evidences for their interaction (known interactions; skyblue-curated database, purple-experimently determined, other predicted interactions; lime-textmining, black-co-expression). Protein of interest studied are enclosed in dotted black circle (**b**) triangle-matrices, where the intensity of color indicates the level of confidence of two proteins which are functionally associated.

To further understand functional overlap among the significantly expressed genes in different pathways, we performed bioinformatic analysis using STRING (v 11.0). The examined genes (*HIF1-α*, *Nrf2*, *VEGF*, *IL1-β*, *CD11b*, *NOTCH1*, *MMP9*, *ERK1*, and *DKK1*) showed interaction among each at the protein level (Figure 7a). Several other proteins were also identified that interact with the studied proteins to modulate specific signaling pathways. Genes differentially expressed in the ERMs of PDR and RD are also shared with the genes belonging to the innate immune system, hypoxia related signaling, inflammatory pathways mediated by microglia, VEGF signaling, *TNF-α* signaling, MAP kinase signaling and extra cellular reorganization (Table 2).

Further, functionally associated proteins involved in the disease pathogenesis were also determined by studying their co-expression. We found MMP9, *IL1-β* and ITGAM (alias for CD11B) are functionally associated and co-expressed. Similarly, *HIF1-α*, and *Nrf2* were co-expressed together showing their strong association in disease pathogenesis (Figure 7b).

## 4. Discussion

Here, we show that activated microglia are a major component of the ERMs from RD and PDR patient samples. Microglial cells are involved in neurotrophic and neurodegenerative disease mechanisms in the retina under stress conditions. In mature retina, microglia reside in the inner and outer plexiform layers and exhibit an abundantly ramified morphology spanning the complete nuclear layers with their long protrusions [21]. Their most important role is to maintain a constant active surveillance of retinal homeostasis where they are indispensable for the immune response and synaptic pruning and transmission [22,23]. Under injury or ischemic stress, they get activated, proliferate and migrate to the site of damage, while releasing pro-inflammatory cytokines and ROS to counter the damage [24]. We recently demonstrated the role of microglial mediated aberrant complement activation in the pathogenesis of DR [25]. That study showed that a gradual increase in the expression of CFH (inhibitor of alternate complement pathway) and CD11b (activated microglial marker) in the retina (at an early stage) culminated in epiretinal membranes changes in DR patients further supporting for a major role of microglia and the alternative complement pathway in disease progression.

While there were not many differences in the cell types present in the membranes from different pathologies, higher number of GFAP positive cells in PDR and RD confirmed the presence of astrocytes and Müller glia and the glial activation (reactive gliosis). Reactive gliosis is known to cause inflammation, which leads to neurodegeneration [26]. The presence of GFAP positive cells could be due to phagocytosis and gliotic responses of Müller glia in the retina. Astrocytes are predominantly localized to the subretinal space near the ILM, where they activate and proliferate during vascular injury and neovascularization. Along with endothelial cells and pericytes, they are also involved in the formation of epiretinal vessels under ischemic condition [27,28]. Besides Müller glia and astrocytes, a large number of activated microglia was also detected in these membranes (RD and PDR). The presence of these cells indicates their role in ERM formation besides underlying gliosis. Such mechanisms may underlie the extensive gliotic changes, which pose surgical challenges.

Activated microglia secrete different types of cytokines [29]. Underlying gliosis causes the induction of inflammation, which in turn promotes oxidative stress. This is supported by our results of immunohistochemistry using anti-OXR1, an oxidative stress responsive gene. The fibrocellular membranes from RD and PDR showed significantly higher number of anti-OXR1 positive cells as compared to MH. Higher expression of CD11b along with significant increase of oxidative stress markers (*HIF1-α* and *Nrf2*), inflammatory markers (*IL1-β* and *MMP9*) and vascular endothelial growth factor (VEGF) in RD and PDR further suggested oxidative stress activates the resident microglia in the retina and adopt inflammatory phenotype. The activated ramified microglia migrate and secrete inflammatory cytokines and growth factors that promote cellular proliferation, neuronal apoptosis and phagocytosis. Inflammatory cytokines could serve as chemoattractants for invading macrophages. Higher expression of VEGF increases the endothelial cell permeability that would leads to further immune cell recruitment and disease manifestation. A study on histopathological analysis of eyes from patients with non-PDR and PDR showed increased numbers of hypertrophic microglia which correlated with disease severity [30].

Activated microglia also express activated C3a and C3b receptors on cell surface and in turn, induce astrocytes to adopt a reactive and proinflammatory phenotype. Such phenomena compromise the ability of neurons to form synapses and phagocytose dead neurons in the central nervous system. Increased deposition of dead neurons could further induce neuroinflammation and neovascularization [31]. We therefore, propose that activated microglia and astrocytes in the ERMs from RD and PDR could result from the proinflammatory phenotype of activated astrocytes in the retina. Further studies are needed to test this hypothesis.

In retinal pigment epithelium cells, Notch2 has been shown to be the major Notch receptor, and its inhibition significantly reduces the intracellular ROS production and cellular apoptosis upon ultraviolet B-induced damage. On the other hand, hypoxic stress to retinal ganglion cells induces Notch1 expression and activation. A study by Jiao et al. 2018, examined the effects of *NOX4* mediated Notch signaling under hyperglycemic (HG) stress. The study observed that HG upregulates Nox4 expression via the activation of Notch signaling, resulting in increased ROS production and cell death in HRECs and thus the inhibition of Notch signaling or Nox4 expression was suggested to be potential therapeutic strategy for the treatment of DR [32].

Several theories have been suggested based on cellular evidences for the processes involved in the membrane formation. Some studies reported that PVR exposes the retina to blood derived fibroblast and other cytokines that causes RPE cells to undergo epithelial to mesenchymal transition and forming proliferative spindle like cells that initiate retinal remodelling and membrane formation over the ILM. Epithelial to Mesenchymal Transition (EMT) facilitates the adoption of a mesenchymal phenotype by an epithelial cell by undergoing multiple biochemical changes. The process of EMT has been well-documented in different types of cancers, leading to tumor progression and increased tumor invasiveness. Besides the other know mechanisms, release of inflammatory cytokines by immune cells has also been shown to cause EMT. Recently, a link between MMP9 and NOTCH1 signalling was also demonstrated [33]. Overexpression of MMP9 acts as a strong activator of NOTCH1 signalling. An earlier study by our group demonstrated increased expression of MMP 9 in PDR vitreous humor that is contributed by activated microglia. The present study also demonstrated an increased expression of CDllb, MMP9, IL-1β and Notch in the membranes as seen by heatmap and co expression using STRING analysis. All together these observations definitely provide a link between increased activation of microglia with RPE undergoing EMT thereby leading to membrane formation as seen in PDR and RD cases by activating Notch 1 signaling and thus inhibiting Notch 1/MMP9 signaling can be a potential therapy to prevent DR severity.

Studies have shown that ERMs impair oxygen and nutrient supply to the retina under ischemic condition [34]. It may also cause tractional retinal detachment by pulling on the neurosensory retina. Our study demonstrated higher level of oxidative stress along with inflammation in RD. Hence, the removal of ERMs may help in improving the oxygenation, nutrient supply and ion transport for retinal potassium buffering [35]. Interestingly, the surgical treatment ILM peeling is found to significantly depend on the type of ERM adherence and area where ERM attaches to the retina. ILM peeling causes mechanical trauma to the retinal nerve fiber layer (RNFL) [36], making this procedure controversial [37].

**Figure 8:**
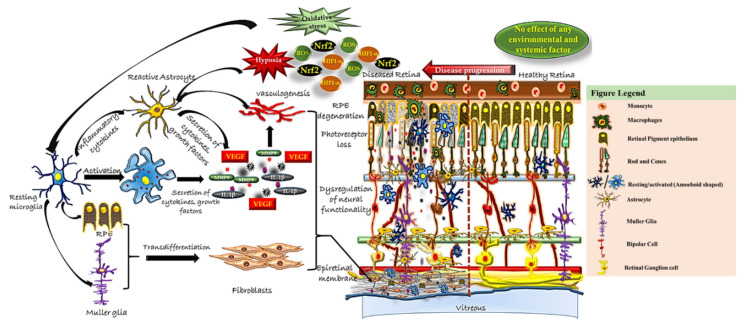
Schematic representation of proposed microglial activity in the epiretinal membrane of the retina. In healthy retina, resident microglia are involved in immune surveillance of all the layers of the retina and maintain retinal homeostatis by synaptic pruning, regulation of neurogenesis and axonal growth. With their phenotypic change of shape, they phagocytose cellular debris. Different kinds of stress/insults lead to abnormal functioning of retinal neurons, microglia, astrocyte, Müller glia and RPE cells. The resident/resting microglia in the retina get activated by transforming into an amoeboid shape and further interact with other neighboring retinal cells, causing abnormal functioning and trans-differentiation into proliferative cells types [10,11]. It also induces the secretion of several chemokines, proinflammatory cytokines and growth factors etc (results from the present study) that could aid in membrane formation by remodeling of the extracellular matrix proteins in retina and thereby contribute to disease pathogenesis.

Our study shows overexpression of oxidative stress-related pathways genes in the fibrocellular membranes (Figure 8). We suggest that modulating the levels of oxidative stress and inflammation in PDR and RD could assist in predicting the need of simultaneous ERM/ILM peeling and may be helpful in improving surgical outcomes.

## 5. Conclusions and Future Scope

We have shown the involvement of microglial cells, inflammation and oxidative stress in fibrocellular membranes (ERM/ILM) of different pathological conditions. Under oxidative stress microglia cells get activated and interact with other glial cells and retinal cells to cause neuroinflammation and neurodegeneration. Reducing oxidative stress and resultant inflammation may help in reducing the risk of ERM formation. Since ILM peeling also has detrimental role on the retina, these findings not only expand our knowledge about ERM pathogenesis but are helpful in identifying newer potential therapies to clinically manage blinding diseases like RD and PDR.

Further investigations on role of microglia and immunomodulation of its phenotype are underway to expand our understanding of its role in DR pathogenesis and better management of this disease and related retinopathies.

## Figures and Tables

**Table 1:** The number of different cell types identified by immunohistochemistry using different specific markers where GFAP; astrocytes/gliotic changes, CRALBP; Müller glia, CD11b; microglia are used.

S. No.	Pathology	H&E	Cell Specific Marker	Mean no. of Positive Cells (*n* = 3) ± SD
1.	MH	No pigmentation	GFAP	0 ± 0
			CRALBP	0 ± 0
			CD11b	0 ± 0
2.	RD	Pigmentation	GFAP	8 ± 8.4
			CRALBP	19 ± 7.0
			CD11b	2.5 ± 3.5
3.	PDR	No pigmentation	GFAP	11 ± 8.7
			CRALBP	6.3 ± 6.6
			CD11b	1.6 ± 1.5

**Table 2:** List of the important pathways involved in ERM pathogenesis.

S. No.	#Term ID	Term Description	Observed Gene Count	Background Gene Count	False Discovery Rate	Matching Proteins in the Network (Labels)
1	hsa04066	HIF-1 signaling pathway	17	98	7.73 × 10^−23^	AKT1,ARNT,CREBBP,CUL2,EGLN1,EGLN2,EP300,FLT1,HIF1A,HMOX1,MAPK3,STAT3,TCEB1,TCEB2,TIMP1,VEGFA,VHL
2	hsa04330	Notch signaling pathway	10	48	5.83 × 10^−14^	CREBBP,EP300,MAML1,MAML2,MAML3,NOTCH1,NOTCH2,NOTCH3,NOTCH4,RBPJ
3	hsa04010	MAPK signaling pathway	14	293	1.23 × 10^−11^	AKT1,DUSP6,FIGF,FLT1,IL1A,IL1B,IL1R1,JUN,KDR,MAPK3,PGF,VEGFA,VEGFB,VEGFC
4	hsa04933	AGE-RAGE signaling pathway in diabetic complications	10	98	2.75 × 10^−11^	AKT1,FIGF,IL1A,IL1B,JUN,MAPK3,STAT3,VEGFA,VEGFB,VEGFC
5	hsa04610	Complement and coagulation cascades	8	78	2.56 × 10^−9^	C3,CD46,CD55,CFB,CFH,CFI,CR1,ITGAM
6	hsa04060	Cytokine-cytokine receptor interaction	11	263	7.33 × 10^−9^	FIGF,FLT1,IL10,IL1A,IL1B,IL1R1,IL1R2,KDR,VEGFA,VEGFB,VEGFC
7	hsa04014	Ras signaling pathway	10	228	2.3 × 10^−8^	AKT1,FIGF,FLT1,KDR,MAPK3,PGF,PTPN11,VEGFA,VEGFB,VEGFC
8	hsa04668	TNF signaling pathway	6	108	6.86 × 10^−6^	AKT1,IL1B,JUN,MAPK3,MMP9,VEGFC
9	hsa04151	PI3K-Akt signaling pathway	9	348	6.87 × 10^−6^	AKT1,FIGF,FLT1,KDR,MAPK3,PGF,VEGFA,VEGFB,VEGFC
10	hsa04310	Wnt signaling pathway	6	143	2.51 × 10^−5^	CREBBP,DKK1,EP300,JUN,LRP5,LRP6
11	hsa04630	Jak-STAT signaling pathway	6	160	4.36 × 10^−5^	AKT1,CREBBP,EP300,IL10,PTPN11,STAT3
12	hsa04370	VEGF signaling pathway	4	59	1.4 × 10^−4^	AKT1,KDR,MAPK3,VEGFA

## Supplementary Materials

The following are available online at https://www.mdpi.com/2076-3921/9/8/654/s1, Table S1: List of antibodies, Table S2: List of qPCR primers, Figure S1: Representative images of ERMs after H&E at 40X showing the glial cells with clear spindle shaped structure of the cells and their long thin processes. Macrophages were also seen with round darkly stained structure. Pigmentation was also observed in the membranes. Figure S2: Characterization of Müller glia cells using AldH1L1 (a specific marker for Müller glia) in MH, iERM, PDR and RD membranes.
