# Supplementary material for: Molecular Assessment of Epiretinal Membrane: Activated Microglia, Oxidative Stress and Inflammation
Source: Antioxidants (Basel). 2020 Jul 23;9(8):654. doi: 10.3390/antiox9080654 (PMC7465764; doi:10.3390/antiox9080654)
Supplement: Supplementary file 1 [file antioxidants-09-00654-s001.pdf]

Supplementary file

Table S1. List of antibodies.

S.No.	Antibodies	Catalog No.
1.	**Anti-GFAP**	Dako Z0334
2.	**Anti-CRALBP**	ab15051, Abcam
3.	**Anti-Aldh1L1**	ab190298, Abcam
4.	**Anti-F4/80**	ab16911, Abcam
5.	**Anti-CD11b**	ab133357, Abcam
5.	**Anti-OXR1**	HPA027395 Sigma
6.	**Anti-Iba1**	ab178680, Abcam
7.	**Alexa Fluor® 488 Goat Anti-Rabbit IgG**	11008-Invitrogen
8.	**Alexa Fluor® 488 Goat Anti-Mouse IgG**	ab150117, Abcam

Table S2. List of qPCR primers

S. No.	Gene	Forward	Reverse
1.	***β-actin***	CATGTACGTTGCTATCCAGGC	CTCCTTAATGTCACGCACGAT
2.	***Hif1-α***	CCAGCAGACTCAAATACAAGAACC	TGTATGTGGGTAGGAGATGGAGAT
3.	***OXR1***	CTGATGGTGATTAAAG ACAGTG	CACTTAAAGACCTCAAACTC C
4.	***NRF2***	AGTGGATCTGCCAACTACTC	CATCTACAAACGGGAATGTCTG
5.	***VEGF165***	ATCTTCAAGCCATCCTGTGTGC	CAAGGCCCACAGGGATTTTC
6.	***CD11b***	CAGACAGGAAGTAGCAGCTCCT	CTGGTCATGTTGATGAAGGTGCT
7.	***MMP9***	TTGACAGCGACAAGAAGTGG	GCCATTCACGTCGTCCTTAT
8.	***DKK1***	GATCATAGCACCTTGGATGGG	GGCACAGTCTGATGACCGG
9.	***ERK1***	CCTGCGACCTTAAGATTTG TGATT	CAGGGAAGATGGGCCGGTTA GAGA
10.	***NOTCH1***	TTGGGAGGAGCAGATTTTTG	CACTGGCATGACACACAACA

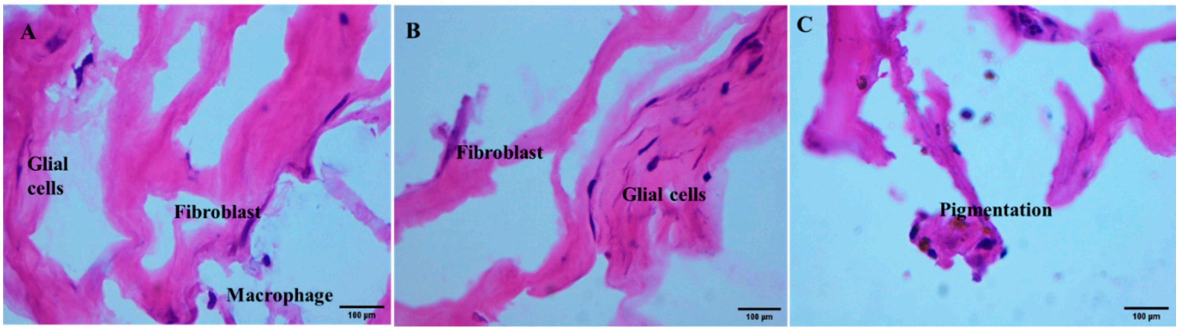

Figure S1. Representative image showing the glial cells with clear spindle shaped structure of the cells and their long thin processes. Macrophages were also seen with round darkly stained structure. Pigmentation was also observed in the membranes.

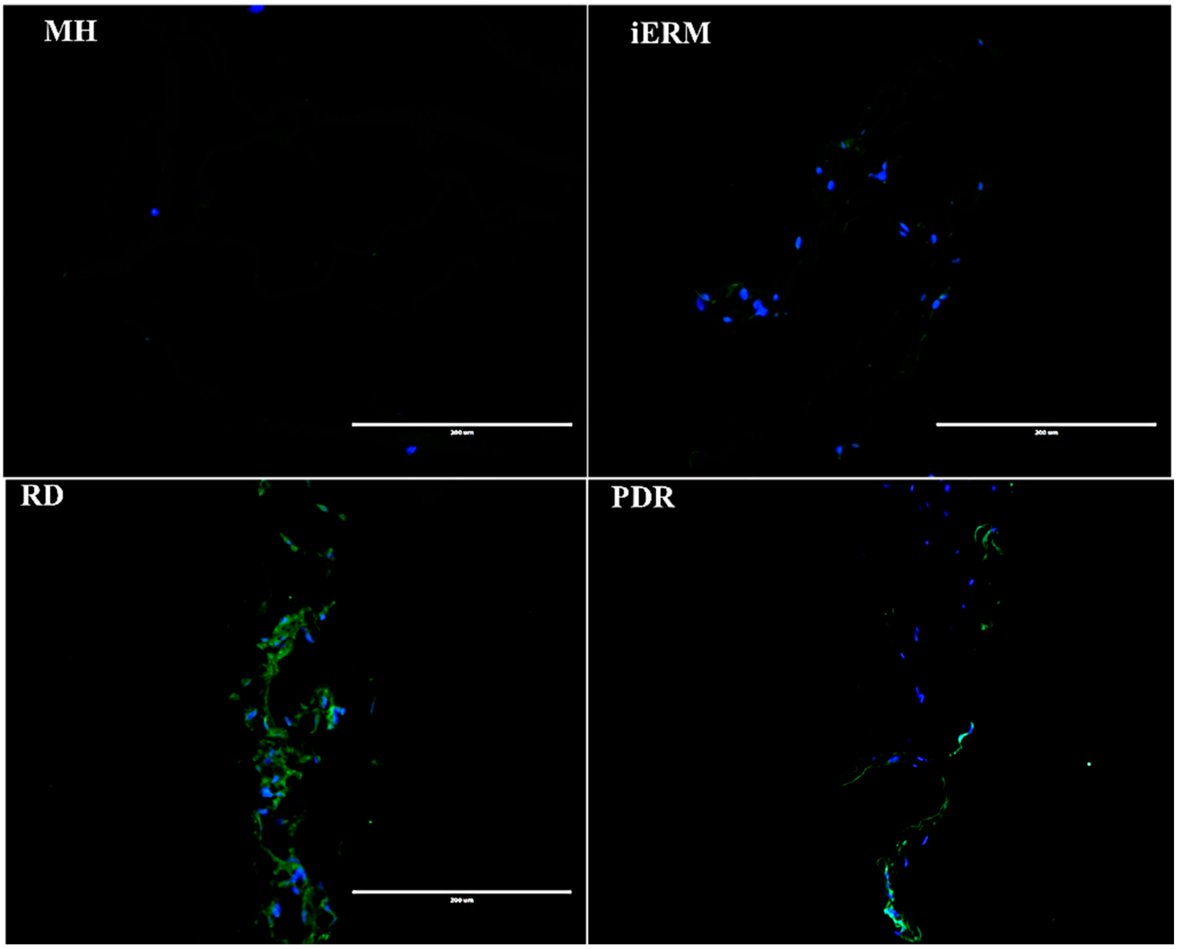

Figure S2. Characterization of Muller glia cells using AldH1L1 (a specific marker for mullerglia) in MH, iERM, PDR and RD membranes.
